# A Survey of Artificial Immune System Based Intrusion Detection

**DOI:** 10.1155/2014/156790

**Published:** 2014-03-23

**Authors:** Hua Yang, Tao Li, Xinlei Hu, Feng Wang, Yang Zou

**Affiliations:** ^1^College of Computer Science, Sichuan University, Chengdu 610064, China; ^2^Computer School, China West Normal University, Nanchong 637002, China

## Abstract

In the area of computer security, Intrusion Detection (ID) is a mechanism that attempts to discover abnormal access to computers by analyzing various interactions. There is a lot of literature about ID, but this study only surveys the approaches based on Artificial Immune System (AIS). The use of AIS in ID is an appealing concept in current techniques. This paper summarizes AIS based ID methods from a new view point; moreover, a framework is proposed for the design of AIS based ID Systems (IDSs). This framework is analyzed and discussed based on three core aspects: antibody/antigen encoding, generation algorithm, and evolution mode. Then we collate the commonly used algorithms, their implementation characteristics, and the development of IDSs into this framework. Finally, some of the future challenges in this area are also highlighted.

## 1. Introduction

Computer security refers to information security as applied to computers and networks, which is an important problem in the world today. This field covers all the processes and mechanisms by which computer based equipment, information and services are protected from unintended or unauthorized access, change, or destruction. With the development of the networks, computer security is facing enormous challenges. To solve this problem, Intrusion Detection Systems (IDSs) have become an indispensable component for detecting abnormal behaviors before they cause widespread damage.

How can we effectively detect all the unauthorized use, misuse, and abuse of computer system? Many researchers have made efforts. Anderson [1] first pointed out the computer Intrusion Detection (ID) problem in 1972. Then he proposed the concept of IDS in 1980 [2] which was one of the earliest works on ID. Between 1984 and 1987, Denning first proposed an IDS model [3]. This prototype was named as the Intrusion Detection Expert System (IDES). 1990 is a watershed in IDS development history. This year, Heberlein developed the Network Security Monitor (NSM) [4]. Then IDS was officially formed as two camps: network based IDS (NIDS) and host based IDS (HIDS). Now, ID is a hot topic in the area of computer security and many prototypes have been developed using different approaches. This paper will discuss various ID methods using Artificial Immune System (AIS).

Computer science has a great tradition of stealing nature's good ideas. The brain has inspired the neural network model which is the basis of many attempts to develop artificial intelligence. The HIS (Human Immune System) is made up of interdependent cell types which protect the body from various harmful pathogenic infections, such as bacteria, viruses, and parasites. It does this largely without prior knowledge of the structure of these pathogens (a more detailed introduction of the HIS can be found in [5, 6]). The goal of HIS is typically referred to as the differentiation of self (molecules and cells that belong to the host organisms) from potentially harmful nonself (molecules and cells that are recognized as foreign molecules). This property has in recent years made it the focus of computer science and ID communities. Hence, applying theoretical immunology and observed immune functions to IDS has gradually developed into a research field called AIS [7]. These years, researchers have made considerable contributions to the development of AIS. A large number of AISs have been built for a wide range of applications including fraud detection [8], optimization [9], machine learning [10], robotics [11], and computer security [12]. Most reviews about AIS based IDS are summarized from the view point of used algorithms or system development. There are so many methods of AIS, which one on earth should we use? Is there any law to follow? This paper will provide a general framework to the area of AIS based IDS and discussion from three aspects: antibody/antigen encoding, generation algorithm, and evolution mode.

In the following sections, we briefly introduce the areas of IDS and AIS.  Section 2 mainly gives the framework for the design of AIS based IDS and introduces the background of AIS. From Section 3 to Section 5, we provide a detailed discussion about the conjunction of IDS and AIS in view of our framework, respectively, antibody/antigen encoding, generation algorithm, and evolution mode. Finally, we present our conclusion and discuss future work of investigation.

## 2. The Framework for the Design of AIS Based IDS

The purpose of the IDS is not only preventing the attack to be happened but also reporting all the abnormal behaviors of the system. In order to design a successful AIS based IDS, the first thing that should be considered is the problem presentation of the system in ID domain and then the combination of AIS methods to IDS. Here, we first introduce AIS briefly. Then, we present the framework design of AIS based IDS.

### 2.1. Background of Artificial Immune System

AIS research began in the mid-1980s with Farmer, Packard, and Perelson's study [13]. Their study suggested that computer science might borrow from the immune system. The great formative AIS researches for computer security were those that proposed the immune system as an analogy for IDSs. One of the classical theories is Negative Selection (NS) [14] which is abstract model of biological NS. In this theory, the detector model generated in censoring phase is intended to monitor the self-state and detect whether or not self has been changed. Then they estimated the method feasibility as a change-detection method on the problem of computer virus detection. Based on the above analysis, Kephart successfully applied immune mechanisms to antivirus problems [15]. With the development of HIS principle, Negative Selection Algorithm (NSA) [14], Clonal Selection Algorithm (CSA) [16], Immune Network Algorithm (INA) [12], and Danger Theory Algorithm (DTA) [17] become the most representative algorithms in the AIS theory. Aickelin et al. [18] provided a detailed overview of immune system approaches to ID. He gave a review of methodologies, algorithms, and research groups in the application of AISs to ID. Kim et al. summarized six immune features that are desirable in an effective IDS [19]. They provided an overview in the view of the research development history.

### 2.2. The Framework for the Design of AIS Based IDS

**Figure 1:**
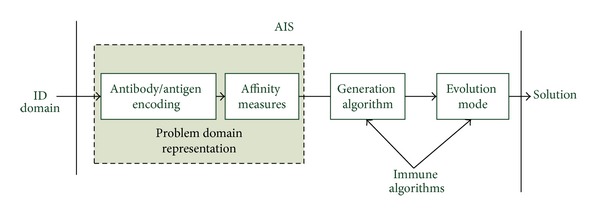
The framework for AIS based IDS design.

Although there are many papers that have summarized the works for this topic, these reviews just divided the current methods into different groups and cannot provide enough guidance information for the design of the AIS based ID methods. In this review, we will introduce these methods from basic elements that a framework for AIS based IDS requires, which are shown in Figure 1.

In order to apply AIS to IDS, three steps are followed in this framework. The first step (the left gray box in Figure 1) is to represent the elements of the system and interaction of individuals in an immune-like form. The goal of this step is to represent the ID elements in an immunology way (e.g., creating abstract models of immune cells, molecules, etc.) and quantify the interaction of these elements by affinity measures. For example the abnormal behavior in IDS is presented as the antigen (nonself) in AIS. In ID domain, affinity means the similarity between detectors and data. Different representations can adopt different affinity measures. The second step is to generate the initial repertoires (generation algorithm), and the third step is to optimize the algorithm (evolution mode). More immune algorithms can be selected for these two steps. This framework can be thought of as a design procedure for engineer AIS inspired IDS. On this foundation three issues will be discussed in the next sections: antibody/antigen encoding, generation algorithm, and evolution mode.

## 3. Antibody/Antigen Encoding

The core of HIS is self and nonself discrimination performed by lymphocytes, which is similar to the IDS that distinguishes normal and abnormal behavior. The key of modeling of this mechanism in AIS based IDS is how to represent the elements in problem domain and decide the matching rules. Antibodies are generated by random combinations of a set of gene segments. Therefore, representation of detectors is to encode them as gene sequences. In AIS based IDS, we follow [12] in assuming the general case that each antibody Ab is a detector represented by an *L*-dimensional vector Ab = 〈Ab_1_, Ab_2_,…, Ab_*L*_〉 and each antigen Ag is a data to be classified which is represented by an *L*-dimensional vector Ag = 〈Ag_1_, Ag_2_,…, Ag_*L*_〉, where *L* is the length of the vector. Each antibody is then matched against each of the antigens and recognized them. The affinity, when mapped into the ID domain, means the similarity between Ag and Ab.

**Figure 2:**
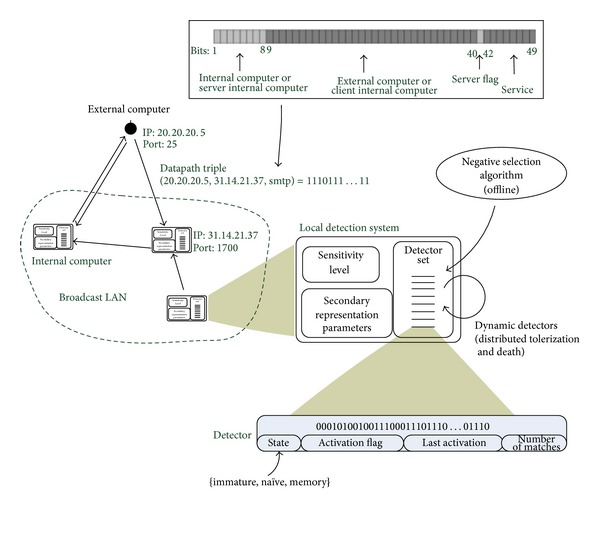
LISYS encoding of a TCP SYN packet [20].

Because any data are eventually implemented as binary bits in computers, researches focused on binary representation as mainstream. That is why binary string is the most commonly adopted coding scheme in AIS. The first AIS model adopted binary encoding, which is suggested by Forrest et al., simulated the self-nonself discrimination principle of the HIS [14]. NSA is the core of this model, by which invalid detectors are eliminated when they matched self data. The NSA adopts binary encoding to simulate antibody/antigen. It breaks 32-bit string into eight substrings as antigen and antibody. Although not many immune features were employed, it shows the feasibility of this algorithm. LISYS (Lightweight Immune SYStem) is a relatively early model system used to protect the LAN from network based attacks [20]. In this system, each detector is a 49-bit binary string, mainly for TCP SYN packet; see Figure 2.

**Figure 3:**
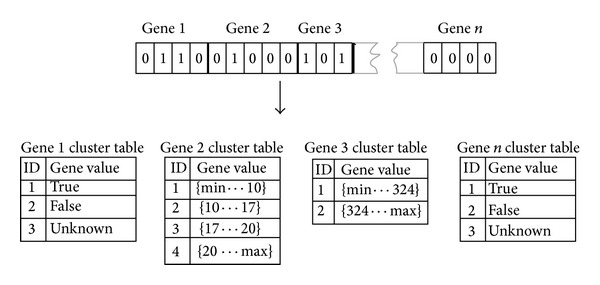
The DynamiCS gene representation [21].

Later, virus-oriented CDIS [23] extended LYSIS further and used 320-bit binary string for each antibody signature, comprising 29 of the possible data fields in a network protocol packet, to detect TCP, UDP, and ICMP. Kim and Bentley used a static CSA with NS operator as one component of the AIS for Network ID (NID). The component was especially developed for the purpose of building a misuse detector in a more efficient way [21]. They use binary genotypes to encode the conjunctive rule detectors, as shown in Figure 3. Then they investigated the dynamic clonal selection, and they found that it can adapt to novel data in NID [24]. A cooperative immunological approach for detecting network anomaly presented set of self as a binary vector for the communication triple (source, destination IP and Port, and protocol) [25].

By changing the encoding from binary to Gray code, the performance can be improved [26]. The reason is that codifications of two consecutive numbers have small Hamming distance. And this method still belongs to the binary encoding.

**Figure 4:**
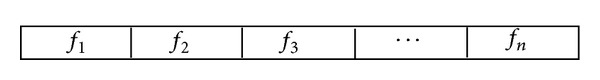
Real-value representation.

Most works have been restricted to binary representation of given data and detectors, but they use different affinity measures, for example, r-contiguous bits matching [14], r-chunks matching [27], landscape-affinity matching [23], Hamming distance [28], and Rogers and Tanimoto (R&T) matching [29], and so forth. However, this antibody/antigen encoding shows several drawbacks. The most significant problem is that the affinity relation between two binary strings represented by the matching rules results in a poor coverage of the problem space [30]. Moreover, the exponential growth of computational time caused by the number of generated detectors is large enough. In order to solve these problems, another different NSA was proposed by Gonzalez et al. [31]. In their method, antibodies were not represented as bit-strings; instead they were represented as hyperspheres. Gonzalez et al. called this approach, real-valued NS; each feature belongs to the range [0.0,1.0] as shown in Figure 4. They focused on real-valued anomaly detection problems rather than ID problems. This algorithm generates hyperspheres with equal radius lengths. Kim used NSA to build an anomaly detector for NID [32]. In the encoding of detectors, each gene of a detector uses decimal notation. The self profile has 33 different fields and this number determines the total number of corresponding genes in the detectors.

In real-valued NS algorithms, a large number of constant-sized detectors are needed to cover large area of nonself space, while no detectors may fit in the small area of nonself space, especially near the boundary between self and nonself [33, 34]. Hence a variable radius was suggested in the variable-sized detectors (termed V-detector) algorithm [35]. V-detector algorithm generates candidate detectors randomly, in which the radius of a detector is dynamically resized until the boundary of the region comes in contact with the nearest hypersphere of a self element. The algorithm terminates if a predefined number of detectors are generated or a predetermined proportion of nonself space is covered. The flexibility provided by the variable radius is easy to realize. Ostaszewski also calculated variable parameters of detectors to cover nonself space [36]. Besides that, a feedback NSA was proposed to solve the anomaly detection, which adjusts adaptively the self and detection radius and the number of detectors according to the detection result [37].

**Figure 5:**
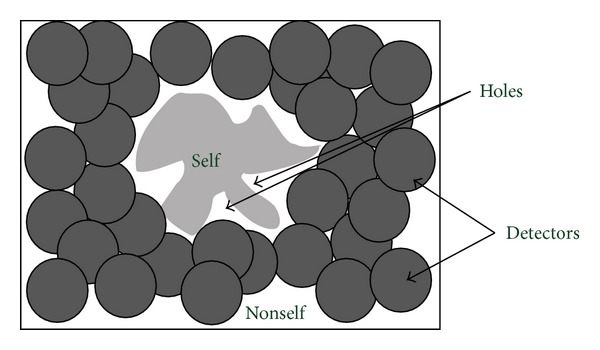
The NSA. Randomly generate candidate detectors (represented by dark circle); if they match any self (i.e., if any of the points covered by the detector are in the self-set), they are eliminated and regenerated until getting enough valid detectors [20].

The issue of holes (the nonself region that cannot be covered by any valid detectors, see Figure 5) induced the geometrical detectors which means that not only the detector radius but also the shape of detector can be changed. Zhou Ji mentioned that detector variability can also be achieved by detector shapes or matching rules and so forth. NS with Detector Rules (NSDR) uses a genetic algorithm to evolve detectors with a hyperrectangle shape that can cover the nonself space. They used a sequential niching technique to evolve multiple detectors in the initial version [38] and then used deterministic crowding as the niching technique in the improved version [39]. In addition, Shapiro et al. used hyperellipsoids instead of hyperspheres to express detectors [40]. Hyperellipsoid is a special hypersphere; it can be stretched and reoriented to fit the boundary of self and nonself. Balachandran et al. incorporated these multiple hypershape detectors together to cover nonself area [41]. Their experimental results demonstrate that multishaped detectors provide better coverage of nonself space than other approaches using only a single type of detectors and less time.

When dealing with real-valued data, the majority of AIS researches use the Euclidean and Manhattan distances on the shape space [42]. Moreover, the difference between Euclidean and Manhattan distances has been discussed by Freitas and Timmis [43]. More information about the other matching rules can be found in [42].

Finally, hybrid representations are possible and intuitively desirable when coping with data sets having attributes of different data types [44]. Numeric attributes are encoded in real-valued format, and category attributes are encoded in strings. In [45], authors chose parameters vector to represent the network pattern, including number of bytes and flag values. Nonetheless, some algorithms cannot handle that data. For instance, [26] apply NSA to a multidimensional personnel data containing both categorical and numeric data. However, instead of using a hybrid categorical/numeric representation and taking all the attributes into account, they simply ignore categorical attributes and work only with numeric attributes.

## 4. Generation Algorithm

Generating accurate and efficient detectors is important when AIS is applied to a detection problem. A good detector must not cover self space and should have minimum overlap with the rest of the detectors. Most NSA based methods randomly generate detectors as described in Forrest's original NSA. Random generation is uniformly distributed among nonself space and resolves problem of unknown nonself space. In training phase, the algorithm randomly generates a set of detectors; each fails to match any element in self. Then in test phase, these detectors are applied to classify new data as self or nonself, like Figure 5.

Although this method is frequently adopted in other research works, as pointed out by Stibor et al. [46], it increases the possibilities of generating invalid detectors. With the increase of self set size, the runtime complexity of detector generation has an exponential growth.

D'haeseleer et al. introduced two detector generating algorithms: linear time detector generating algorithm and greedy detector generating algorithm [47]. They were compared with the Forrest method which is called “exhaustive detector generating algorithm.” The linear algorithm solves a counting recurrence for the number of strings unmatched by strings in candidate detectors and then uses the enumeration imposed by the counting recurrence to pick detectors randomly from this set of candidate detectors. Compared to the exhaustive algorithm, the advantage of linear algorithm is obvious, because it removes the pattern strings which will not become valid detector strings. The greedy algorithm improves upon the linear algorithm through the elimination of redundant detectors. It spreads the detectors apart and provides the maximum coverage for a given number of detectors. Nevertheless it sacrifices the speed of detector generation; the time will increase linearly with the size of self set. Castro and Timmis proposed the NS with mutation algorithm (NSMutation) which has better performance in terms of time complexity. NSMutation has a slight modification of the exhaustive stage of the NS by introducing somatic hypermutation [12]. The goal of NSMutation algorithm is to guide the candidate detector away from self set during the process of mutating a candidate detector. In [48], the authors drew a conclusion that NSMutation is similar to the exhaustive algorithm with the difference of eliminating redundancy and possessing parameters that can be optimized for better performance. All these detector generating algorithms time and space complexities are shown in Table 1, where *m* is the alphabet cardinality, *l* is the string length, *r* is matching threshold, *N*_*S*_ is the number of self, and *N*_*R*_ is the number of detectors.

In HIS, clonal selection is used to proliferate and differentiate the stimulation of cells with antigens. Burne proposed in 1959 [49] that we can improve the random detector generation by clonal selection principle. The artificial form of clonal selection was popularized by de Castro and Von Zuben. They gave an algorithm called CSA [50], which was then modified and renamed as CLONALG [9]. Garrett introduced an adaptive CSA as a modification of CLONALG [51]. CSA has always been used as strategy towards optimization and pattern recognition [52]. It is a colony search mechanism in nature, which enables detectors to clone their parents according to a mutation mechanism with high rates. This strategy evolves the immune systems so that they can deal with antigens that it has encountered in the past. From this, researchers combine clonal selection with other methods to solve ID problems. Kim and Bentley adopted the clonal selection as one component of the AIS for NID [25, 26, 52]. Liu et al. applied the CSA to the process of modeling normal behavior in ID, and experimental results showed that the algorithm has higher detection rate (DR) and lower false alarm rate (FA) [53], compared with the algorithms which apply the genetic algorithm to ID or apply the NSA of the AIS to ID. Tang et al. presented an avidity model based CSA for NID, which also has higher DR and lower FA compared with other approaches [54]. Besides that, many other approaches were mentioned in [55]. Additionally, the famous immune network model aiNet [56] also uses CLONALG with added network interactions. The mechanism used by the aiNet model is based on the ideas of clonal selection, and it mainly combines with the immune network theory. A network of stimulatory and suppressive interactions exists between antibodies that affects the concentrations of each type of antibody and then reaches a state of equilibrium. For more information, please refer to [57].

According to the features of AIS, many methods and techniques have been combined with AIS to better detect the abnormal behavior, like artificial neural networks, fuzzy systems, and genetic algorithms. For instance, [31] combined NSA and a conventional classification algorithm to perform anomaly detection; [58] presents an immunofuzzy approach to anomaly detection, because fuzzy logic can provide a better definition of the boundary between normal and abnormal behavior; Dasgupta et al. proposed a Multilevel Immune Learning Algorithm (MILA) to detect intrusions and issue alarms [59]. MILA detection used multiple strategies to generate detectors, where T detectors performed a low-level continuous bitwise match, while the B detectors performed a high-level match at noncontiguous positions of strings. Activated T detectors will further provide a signal to help activate B detectors. This model further simulated NSA, CSA, and somatic hypermutation of mature T cells and B cells. A hybrid system composed of AIS and self organising map is presented in [60]. Their experimental results showed higher detection and classification rate for Denial-of-Service and User-to-Root attacks.

Self and nonself discrimination is the fundamental principle which guides the AIS development. Therefore, NS acts as an important role in AIS. However, Matzinger proposed the Danger Theory (DT) and claimed that immune responses are triggered by the danger signals that are sent out when cells die an unnatural death, not by nonself antigens [61, 62]. It provides a fresh idea for AIS. Based on this idea, Aickelin and his research group applied DT to IDSs [17, 63]. In their research, danger signals are represented as numbers. Then, Twycross and Aickelin presented a libtissue framework incorporating ideas from innate immunity into AISs. The libtissue has a client/server architecture. Clients in libtissue collect antigen and external signals and transmit them to the libtissue server. The servers implement the AIS algorithm. They used libtissue for dynamic anomaly detection. From the dendritic cells and their interaction with T cells of the DT, the Dendritic Cell Algorithm (DCA) and Toll-Like Receptor Algorithm (TLRA) were proposed by Greensmith and Aickelin, Twycross and Aickelin, respectively. The DCA plus libtissue framework can scan port [64, 65]. The TLRA was deployed in the libtissue framework to detect process anomaly [66, 67]. Nonetheless, the DCA relies on the signal processing aspect by using multiple input and output signals, while the TLRA only uses danger signals. But the DTA is still controversial among immunologists about how to clearly define the danger signals.

## 5. Evolution Mode

**Figure 6:**
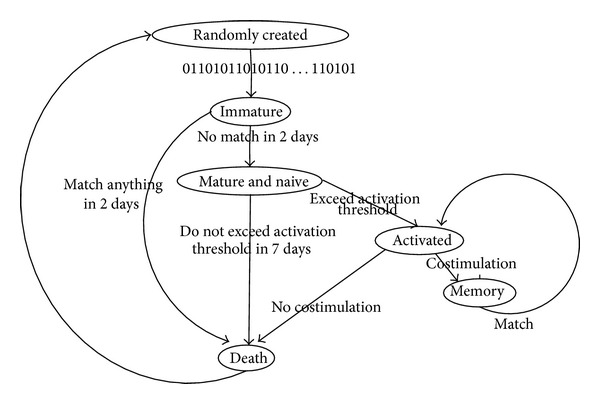
The lifecycle of a detector [20].

With the development of the system, the detectors will increase. However, the system is finite, like the body; we cannot generate detectors infinitely. The old and invalid detectors must be eliminated. Whilst the intrusion behaviors appear every day, the new detectors must generate and evolve to detect them. Instead of inefficiently throwing away detectors that match self samples, Hofmeyr suggested changing the detectors over time, that is, to make them dynamic [20]. He gave each detector a finite lifetime; at the end of lifetime, the detector will be eliminated and replaced by a new randomly generated detector. He gave a figure of the lifecycle for a detector as shown in Figure 6.

Ayara et al. [48] and González and Dasgupta [68] tried to give detectors a period of time before eliminating them. Kim and Bentley investigated a further extension of DynamiCS [69]; when memory detectors show a poor degree of self-tolerance to new antigens, they will be eliminated. Li proposed a receptor editing inspired real NSA [70]. For the detector that matches self, algorithm uses directional receptor editing to make a new life, and, for the detector that does not match self, algorithm uses direction receptor editing for identifying identical nearest self to expand coverage of nonself space.

If new detectors are generated by taking some feedback from previous detectors instead of random, then the new detector can be better suited for the nonself antigens. Hightower et al. [71], Perelson et al. [72], and Oprea and Forrest [73] employed a Genetic Algorithm (GA) to study the effects of evolution in the genetic encoding of the antibody molecules, which can be seemed as a feedback strategy. Moreover, in [74] Kim and Bentley embedded gene library evolutionary stage in their artificial immune model for NID. The gene library is a dynamic evolutionary library which stores the potential genes of detectors and diverse genetic mechanisms generate new detectors. The potential genes are the selected fields of profiles to describe anomalous network traffic patterns. After that, they use deleted memory detectors as the virtual gene library [75]. In fact, their method is consistent with the HIS theory, because deleted detectors also come from gene libraries. Zeng also uses gene library to generate the new detectors in initial IDS [76]. Thus, gene libraries provide a way of remembering past encounters so that antibody creation is more likely to match novel clusters which are nevertheless similar to those seen some time ago. More information about evaluation of the gene libraries in the AIS can be found in [77].

Gene library is an approach which guides the generation process to create antibodies with a good probability of success. However, gene library approaches are relatively complex. In addition to changing the radius and shape of the detector, another approach to improve the effectiveness is just moving the position of the detector. González and Dasgupta calculated the k-nearest neighbors of detector in the self set, and then the median distance of these k-neighbors is computed. If this median distance is less than a threshold, the detector is considered to match self and moves away to the opposite direction. This strategy is good to be robust to noise and outliers [68]. Laurentys et al. allocated the detectors in nonself space mixed moving detectors and generating detectors with constant radius and V-detector together [78].

**Figure 7:**
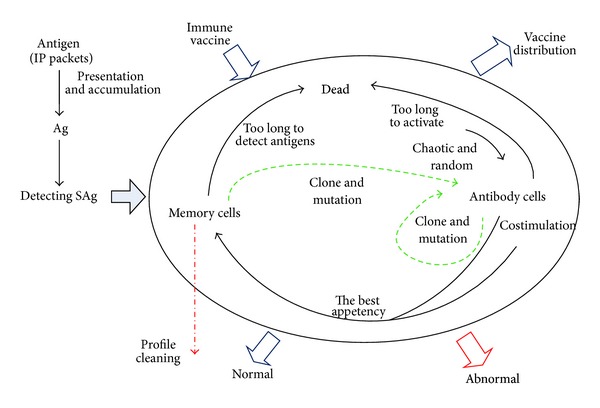
Dynamical real-time anomaly detection with immune NS [22].

An IDS evaluates a suspected intrusion once it has taken place and signals an alarm. In fact, most current ID methods cannot process large amounts of audit data for real-time operations. The roles of self and nonself may dynamically exchange; that is, the legal behaviors this time may be dangerous the next time, and vice versa. In the past few years, computer scientists have designed immune inspired algorithms that could detect the abnormal behavior effectively. DynamiCS has done a trial on this situation [24]. It can be able to deal with a real environment where self behaviors change after a certain period. DynamiCS introduced three important parameters: tolerization period of an immature detector, activation threshold of a mature detector, and the life span of a mature detector, but only one detection period for the self updating; it is too short to collect enough self elements. Li proposed a new immune based dynamic ID (Idid) model [79]. In Idid, the dynamic models and the corresponding recursive equations of the lifecycle of mature lymphocytes and the immune memory are built; the self and nonself dynamic description is solved. Yang et al. presented a model of network security based on AIS which utilized distributed agents to capture the network traffic in real time [80]. The model depicted the dynamic evolutions of self, antigens, immune-tolerance, lifecycle of mature agent, and immune memory. Their experimental results show that it has the features of real-time processing and self-adapting. Peng et al. proposed a Dynamic Anomaly Detection Algorithm with Immune NS (DADAI) [22], combining the antibody's clone theory and vaccination. It established dynamic evolvement formulations of detection profiles which can dynamically synchronize detection profiles with the real network environment. The algorithm is contained in Figure 7. Theoretical analysis and experimental results showed that DADAI can be effectively deployed on the real-time NID under high-speed network environment.

## 6. The Future of Intrusion Detection

This review concentrated on the AIS based IDS. It first presented a brief introduction to the AIS in order to provide the readers with the background to understand. The main contribution of this paper is the framework for the design of AIS based IDS. Based on the framework, three aspects were described, followed by explorations of the literatures about IDSs. These theories and approaches based on AIS are able to combine to serve as a base for effective ID through our analysis. From the analysis of our framework, we find that system with real-valued representation is better suited for IDS, in which detectors effectively generate and dynamically evolve.

In the more recent years, AIS research has drifted away from more biologically appealing models to biological details, such as DCA, which is inspired by the role of dendritic cells (a specialized antigen presenting cells that provide a vital link between the innate and adaptive immune system) [81]. It is more useful in computer security, as not all abnormal events represent attacks [65, 82]. Grossman's Tunable Activation Threshold (TAT) hypothesis [83] is another perspective. TAT posits that each individual immune cell has its own tunable activation threshold whose value reflects the recent history of interactions with the surrounding environment. Antunes and Correia [84] described the deployed TAT based AIS for NID; [85] gives the analysis of TAT model. There are many useful and powerful algorithms that have already arisen and can arise when more than two of the different approaches are hybridized or new HIS theory is proposed.

Like [85] and [82–84], many summaries of the research in AIS were reported. HIS embodies the features of robustness, distribution, lightweight, self-organizing, and self-adapting. AISs are highly abstract models of their biological counterparts applied to solve problems in different areas. The analogy between the HIS and IDS naturally attracts computer scientists to make research on immune system approaches to ID. AISs have also been used in conjunction with other approaches in order to create more powerful models and improve individual performances.

Despite the existing advantages of AIS, now IDSs still have many problems, for example, lack of support of IPv6 addressing scheme, high levels of false positive and false negative alarm rates, lack of quick response for the unknown attacks. And AIS is a relatively young field; AIS based IDS faces many difficulties: real-world environments are much more complicated, self set constantly changes, and detection is in real time. In order to resolve all these issues and make progress for this research, our future IDSs should focus on the questions of quick response and less false alarm and false negative. In the future, depending on the biological immune mechanism, it will be able to propose effective ID models and algorithms, although there will be a difficult and winding road.

## Figures and Tables

**Table 1:** Time and space complexities of all detector generating algorithms [48].

Algorithm	Time	Space
Exhaustive	*O*(*m* ^*l*^ · *N*_*S*_)	*O*(*l* · *N*_*S*_)
Linear	*O*((*l* − *r* + 1) · *N*_*S*_ · *m* ^*r*^) + *O*((*l* − *r* + 1) · *m* ^*r*^) + *O*(*l* · *N*_*R*_)	*O*((*l* − *r* + 1)^2^ · *m* ^*r*^)
Greedy	*O*((*l* − *r* + 1) · *N*_*S*_ · *m* ^*r*^) + *O*((*l* − *r* + 1) · *m* ^*r*^ · *N*_*R*_)	*O*((*l* − *r* + 1)^2^ · *m* ^*r*^)
NSMutation	*O*(*m* ^*l*^ · *N*_*S*_) + *O*(*N*_*R*_ · *m* ^*r*^) + *O*(*N*_*R*_)	*O*(*l* · (*N*_*S*_ + *N*_*R*_))
